# Clinical and laboratory characteristics associated with referral of hospitalized elderly to palliative care

**DOI:** 10.1590/S1679-45082018AO4092

**Published:** 2018-04-06

**Authors:** Suelen Pereira Arcanjo, Luis Alberto Saporetti, José Antonio Esper Curiati, Wilson Jacob-Filho, Thiago Junqueira Avelino-Silva

**Affiliations:** 1 Hospital das Clínicas, Faculdade de Medicina, Universidade de São Paulo, São Paulo, SP, Brazil; 2 Faculdade de Medicina, Universidade de São Paulo, São Paulo, SP, Brazil

**Keywords:** Aged, Palliative care, Hospital care, Prognosis, Decision making, Clinical decision-making, Idosos, Cuidados paliativos, Assistência hospitalar, Prognóstico, Tomada de decisões, Tomada de decisão clínica

## Abstract

**Objective:**

To investigate clinical and laboratory characteristics associated with referral of acutely ill older adults to exclusive palliative care.

**Methods:**

A retrospective cohort study based on 572 admissions of acutely ill patients aged 60 years or over to a university hospital located in São Paulo, Brazil, from 2009 to 2013. The primary outcome was the clinical indication for exclusive palliative care. Comprehensive geriatric assessments were used to measure target predictors, such as sociodemographic, clinical, cognitive, functional and laboratory data. Stepwise logistic regression was used to identify independent predictors of palliative care.

**Results:**

Exclusive palliative care was indicated in 152 (27%) cases. In the palliative care group, in-hospital mortality and 12 month cumulative mortality amounted to 50% and 66%, respectively. Major conditions prompting referral to palliative care were advanced dementia (45%), cancer (38%), congestive heart failure (25%), stage IV and V renal dysfunction (24%), chronic obstructive pulmonary disease (8%) and cirrhosis (4%). Major complications observed in the palliative care group included delirium (p<0.001), infections (p<0.001) and pressure ulcers (p<0.001). Following multivariate analysis, male sex (OR=2.12; 95%CI: 1.32-3.40), cancer (OR=7.36; 95%CI: 4.26-13.03), advanced dementia (OR=12.6; 95%CI: 7.5-21.2), and albumin levels (OR=0.25; 95%CI: 0.17-0.38) were identified as independent predictors of referral to exclusive palliative care.

**Conclusion:**

Advanced dementia and cancer were the major clinical conditions associated with referral of hospitalized older adults to exclusive palliative care. High short-term mortality suggests prognosis should be better assessed and discussed with patients and families in primary care settings.

## RESUMO

**Objetivo:**

Investigar as características clínicas e laboratoriais associadas à indicação de cuidados paliativos exclusivos em idosos gravemente enfermos.

**Métodos:**

Neste estudo retrospectivo de coorte, foram incluídas 572 admissões de pacientes gravemente enfermos com idade igual ou superior a 60 anos, em um hospital universitário, em São Paulo, no período de 2009 a 2013. O desfecho primário foi a indicação clínica de cuidados exclusivamente paliativos. Empregamos avaliações geriátricas amplas padronizadas para mensurar preditores de interesse, incluindo informações sociodemográficas, clínicas, cognitivas, funcionais e laboratoriais. Utilizamos modelos de regressão logística *stepwise* para identificar preditores independentes de cuidados paliativos.

**Resultados:**

Foram indicados cuidados exclusivamente paliativos em 152 (27%) casos. No grupo de cuidados paliativos, a mortalidade hospitalar foi de 50%, sendo que a mortalidade acumulada em 12 meses atingiu 66%. Os diagnósticos mais frequentemente apontados como motivadores de cuidados paliativos foram demência avançada (45%), câncer (38%), insuficiência cardíaca congestiva (25%), insuficiência renal estágios 4 e 5 (24%), doença pulmonar obstrutiva crônica (8%) e cirrose (4%). As complicações mais comuns no grupo de cuidados paliativos foram delírio (p<0,001), infecções (p<0,001) e úlceras por pressão (p<0,001). Após análise multivariada, sexo masculino (OR=2,12; IC95%: 1,32-3,40), câncer (OR=7,36; IC95%: 4,26-13,03), demência avançada (OR=12,6; IC95%: 7,5-21,2) e níveis de albumina (OR=0,25; IC95%: 0,17-0,38) foram identificados como preditores independentes da indicação de cuidados exclusivamente paliativos.

**Conclusão:**

Demência avançada e câncer foram as condições clínicas mais frequentemente associadas à indicação de cuidados paliativos exclusivos em idosos hospitalizados. A elevada mortalidade a curto prazo sugere que o prognóstico deva ser melhor avaliado e discutido com os pacientes e suas famílias no ambiente de Atenção Primária.

## INTRODUCTION

Population aging and advances in modern medicine have led to significant increases in chronic and degenerative disease prevalence. Given many of these conditions are incurable, individual prognosis must be carefully considered in clinical decision-making and quality of life prioritized in care management.^(^ ^1^ ^)^

Palliative medicine aims to fulfill the needs of patients suffering from life-threatening diseases and relieving physical, psychosocial and spiritual suffering.^(^ ^2^ ^)^ Yet, palliative care should not be limited to end-of-life care, but provided to patients over the course of their illnesses to optimize benefits. It is estimated that 40 million people worldwide require palliative care at any point in time, with progressive nonmalignant diseases accounting for the highest proportion of patients, followed by cancer.^(^ ^3^ ^)^ Even data from developing countries, such as Brazil, indicate that chronic degenerative conditions are more frequently associated with palliative care eligibility.^(^ ^4^ ^)^ This is particularly true for older adults who experience physical and functional decline over the course of illness, and often die of complications from protracted conditions, such as dementia, heart failure and kidney disease.^(^ ^5^ ^-^ ^7^ ^)^

In this context, advanced care planning should help patients prepare for treatment-related decision making, preferably based on personal values and riskbenefit assessments of available alternatives. However, a consensus on how to best assess prognosis in geriatric patients is lacking, and timely transition from therapeutic to palliative care is challenging.^(^ ^8^ ^,^ ^9^ ^)^ As a result, palliative care is often delayed in older adults and non-cancer patients,^(^ ^10^ ^)^ and some important factors, such as functional, cognitive and nutritional status, are commonly underestimated. Therefore, clinicians could benefit from identification and communication of red flag symptoms suggestive of clinical deterioration.^(^ ^7^ ^)^

Given this population is often hospitalized, and studies indicate that up to 36% of inpatients meet palliative care criteria, hospitals should seize the opportunity to evaluate such patients and plan their clinical care accordingly, based on established criteria and measures.^(^ ^11^ ^)^

## OBJECTIVE

To investigate clinical and laboratory characteristics associated with referral of acutely ill older adults to exclusive palliative care, to determine associated 12 month survival rates, and to investigate patient and family participation in the decision-making process.

## METHODS

Prospective cohort study based on consecutive admissions of acutely ill older adults to the geriatric ward of a tertiary university hospital located in São Paulo, Brazil, from January 2009 to August 2013.

This geriatric unit had 18 beds and received nonsurgical and non-orthopedic inpatients aged 60 years or over. Patients were followed by a multidisciplinary team consisting of geriatricians, nurses, physical therapists, audiologists and speech therapists, social workers, psychologists and dietitians. Age was the sole formal criterion for admission; however, the unit was in fact a specialty ward preferably admitting high complexity, high vulnerability older adults referred from the emergency department. Patients already under palliative care upon admission and cases with missing data regarding the main covariates were excluded from the analysis.

Data were extracted from comprehensive, standardized geriatric assessments completed for all hospitalizations within the first 24 hours of admission.^(^ ^12^ ^)^ Assessments were performed by trained fellow geriatricians and supervised by permanent staff of geriatritians.

The primary outcome was the clinical indication for referral to exclusive palliative care in the geriatric ward, according Gold Standards Framework principles.^(^ ^13^ ^)^ Upon patient's death or discharge, attending physicians recorded whether palliative care had been implemented over the course of hospital stay. Moreover, they entered data on clinical factors contributing to referral to exclusive palliative care; existence of family/caregiver meetings; patient awareness of their own diagnosis and prognosis; prescription of opioids for symptom relief; and prescription of terminal sedation.

Admission assessments included detailed information on clinical diagnosis, socio-demographic characteristics, prescriptions, cognitive and functional status, physical examination and laboratory tests.^(^ ^14^ ^,^ ^15^ ^)^ Functional status measurement was based on six activities of daily living (ADLs) and nine instrumental activities of daily living (IADLs).^(^ ^16^ ^,^ ^17^ ^)^ Activities of daily living and IADLs were scored from zero to 2, with higher scores representing greater independence.^(^ ^18^ ^)^ Dementia severity was staged according to the Clinical Dementia Rating scale (CDR).^(^ ^19^ ^)^ Delirium was defined according to Confusion Assessment Method (CAM) criteria applied upon admission and over the course of hospital stay.^(^ ^20^ ^,^ ^21^ ^)^ Polypharmacy was defined as chronic use of five or more drugs. Comorbidity and disease burdens were determined using the Charlson et al., comorbidity index and the Burden of Illness Score for Elderly Patients (BISEP), respectively.^(^ ^15^ ^,^ ^22^ ^)^ Further follow-up data regarding new diagnoses and complications, functional status and hospitalization summary were collected upon death or discharge.

In-hospital death was recorded at the end of hospital stay. Twelve-month mortality data were collected via telephone contacts with patients or caregivers, or based on hospital management records (unsuccessful contact attempts).

The study was approved by the Human Research Ethics Committee under protocol number 368.073, CAAE: 18474613.5.0000.0068. Patient identifiable information was stored in locked cabinets and/or secure electronic servers.

### Statistical analysis

Descriptive analysis of demographic, clinical and laboratory features was based on means and standard deviations, medians and interquartile ranges (IQR), counts and proportions. Categorical variables were compared in each group using the χ^2^ test. Continuous variables were compared using the Student's *t* test or the Wilcoxon rank-sum test as appropriate. Independent predictors of palliative care were investigated using backward elimination and stepwise logistic regression (retention of covariates with p value <1). Survival was plotted in Kaplan-Meyer curves and tested with log-rank tests. Statistical tests were two-tailed and an alpha error of up to 5% was accepted. Statistical analyses were performed using Stata 14 (Stata Corp, College Station, Texas, USA).

## RESULTS

This study involved 572 cases from an initial sample of 624 consecutive admissions of acutely ill older adults. Cases excluded corresponded to patients already under exclusive palliative care upon admission (15 cases; 2%) or incomplete assessments (37 cases; 6%). Missing data were assumed to be non-preferential and occurred mostly at the beginning of the year, during the 4 weeks corresponding to the comprehensive geriatric assessment model training period.

Mean age of patients in this sample was 81 years, and 63% of patients were women. Overall in-hospital mortality amounted to 21%, with a cumulative mortality of 39% in 12 months. Most patients had less than 4 years of education (69%) and declared a religious affiliation (75%). A total of 53% of the cases had Charlson comorbidity index of four or higher, while 57% had group IV BISEP score. Cancer was diagnosed in 98 (17%) patients, of whom 40 had metastatic lesions. Upon admission, almost half the patients (47%) were considered totally dependent in ADLs and IADLs and 37% presented with moderate to severe dementia. Patients with chronic kidney disease, cerebrovascular disease, cancer, advanced dementia, delirium, polypharmacy, weight loss history, functional dependency, pressure ulcers and low albumin levels were more likely to die over the course of follow-up (Table 1).

**Table 1:** Population characteristics associated with in-hospital and 12 month mortality

Characteristics		Total (n=572)	In-hospital death			12 month death (discharged patients)		
			No (n=450)	Yes (n=122)	p value	No (n=350)	Yes (n=100)	p value
Demographics								
	Age, years (SD)	81 (±8)	81 (±8)	82 (±9)	0.185	80 (±8)	82 (±8)	0.884
	Women, n (%)	362 (63)	288 (64)	74 (61)	0.497	230 (66)	58 (58)	0.156
	Married, n (%)	210 (37)	158 (35)	52 (43)	0.127	118 (34)	40 (40)	0.245
Comorbidities, n (%)								
	Heart failure	178 (31)	142 (32)	36 (30)	0.665	104 (30)	38 (38)	0.116
	Cerebrovascular disease	120 (21)	100 (22)	20 (16)	0.161	64 (18)	36 (36)	<0.001
	COPD	72 (13)	58 (13)	14 (11)	0.676	44 (13)	14 (14)	0.707
	Chronic kidney disease	146 (26)	102 (23)	44 (36)	0.003	58 (17)	44 (44)	<0.001
	Depression	144 (25)	118 (26)	26 (21)	0.268	94 (27)	24 (24)	0.567
	Cancer*	98 (17)	54 (12)	44 (36)	<0.001	44 (13)	10 (10)	0.485
	Charlson, median (IQR)	4 (2,6)	4 (2,5)	4 (2,7)	0.062	3 (3,5)	4 (3,6)	<0.001
Geriatric syndromes, n (%)								
	Polypharmacy	266 (47)	198 (44)	68 (56)	0.021	168 (48)	30 (30)	0.001
Activities of daily living								
	Independent	127 (22)	103 (23)	24 (20)	0.029	20 (71)	32 (32)	0.001
	Partially dependent	176 (31)	148 (33)	28 (23)		130 (37)	18 (18)	
	Totally dependent	269 (47)	199 (44)	70 (57)		149 (43)	50 (50)	
	Advanced dementia*	128 (22)	102 (23)	26 (21)	0.75	68 (19)	34 (34)	0.002
	Delirium*	230 (40)	150 (33)	80 (66)	<0.001	106 (30)	44 (44)	0.010
	Pressure ulcers	66 (12)	44 (10)	22 (18)	0.011	28 (8)	16 (16)	0.018
	Weight loss	160 (28)	106 (24)	54 (44)	<0.001	88 (25)	18 (18)	0.138
Laboratory tests								
	Hemoglobin, g/dL (SD)	11.3 (±2.4)	11.3 (±2.4)	11.2 (±2.4)	0.644	110.5 (±20.4)	100.9 (±20.1)	0.023
	GFR, mL/min (IQR)	45 (30,65)	47 (31,64)	41 (22,74)	0.312	50 (35,65)	31 (23,52)	<0.001
	Albumin, g/dL (SD)	3.3 (±0.6)	3.4 (±0.6)	3.0 (±0.6)	<0.001	30.5 (±00.6)	30.1 (±00.5)	<0.001
	Palliative care recommendation	152 (27)	76 (17)	76 (62)	<0.001	52 (15)	24 (24)	0.031

* data consolidated at discharge.

SD: standard deviation; COPD: chronic obstructive pulmonary disease; IQR: interquartile range.

Exclusive palliative care was indicated over the course of hospital stay in 152 (27%) cases (median of 4 days after admission; IQR: 1.7). Family/caregiver meetings preceded formal implementation of palliative care in 89% of cases; yet, according to attending physicians, only 11% of 70 patients eligible for palliative care and without significant cognitive impairments were aware of their prognosis. Opioids were prescribed in 49% cases eligible for palliative care and terminal sedation employed in 5% of in-hospital deaths.

Major clinical factors associated with referral to palliative care were advanced dementia (45%), cancer (38%), congestive heart failure (25%), stage IV and V kidney failure (24%), chronic obstructive pulmonary disease (8%) and cirrhosis (4%). Common complications in palliative care patients included delirium (p<0.001), infections (p<0.001) and pressure ulcers (p<0.001). Multivariate analysis revealed male sex, cancer, advanced dementia and low serum albumin levels to be independent predictors of referral to exclusive palliative care (Table 2).

**Table 2:** Predictors of palliative care recommendation in hospitalized older adults

		Palliative care (%)	Unadjusted odds ratios	Adjusted* odds ratios	Adjusted p value
Age, 10 years		-	1.02 (0.82-1.28)	-	-
Sex					
	Female	22	Ref.	Ref.	Ref.
	Male	34	1.84 (1.26-2.68)	2.12 (1.32-3.40)	0.002
Married					
	Yes	30	Ref.	-	-
	No	25	0.79 (0.54-1.16)		
Heart failure					
	No	20	Ref.	-	-
	Yes	29	1.64 (1.08-2.52)		
Cerebrovascular disease					
	No	25	Ref.	-	-
	Yes	33	1.52 (0.98-2.35)		
COPD					
	No	28	Ref.	-	-
	Yes	24	0.63 (0.34-1.17)		
Chronic kidney disease					
	No	27	Ref.	-	-
	Yes	26	0.96 (0.63-1.48)		
Cancer					
	No	22	Ref.	Ref.	Ref.
	Yes	51	3.80 (2.42-5.97)	7.36 (4.16-13.03)	<0.001
Advanced dementia					
	No	17	Ref.	Ref.	Ref.
	Yes	61	7.8 (5.05-12.04)	12.6 (7.5-21.2)	<0.001
Activities of daily living					
	Independent	16	Ref.	-	-
	Partially dependent	15	0.97 (0.52-1.82)		
	Totally dependent	39	3.43 (2.0-5.86)		
Albumin, g/dL		-	0.31 (0.22-0.43)	0.25 (0.17-0.38)	<0.001

* backwards stepwise logistic regression.

Ref.: reference category; COPD: chronic obstructive pulmonary disease.

**Figure 1:**
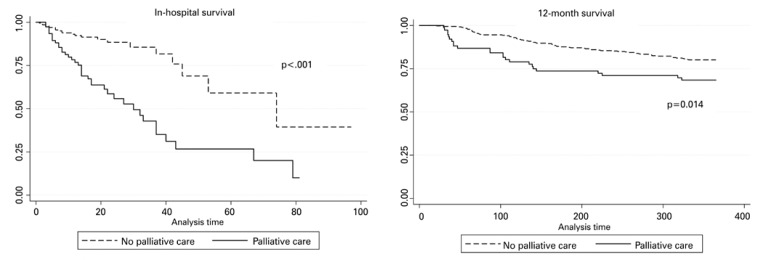
Kaplan-Meier survival estimates according to palliative care recommendation and corresponding log-rank tests

In the palliative care group, in-hospital and cumulative 12 month mortality amounted to 50% and 66%, respectively. The unadjusted relative risk of death associated with referral to palliative care was 4.57 (95% confidence interval − 95%CI: 3.33-6.26; p<0.001) for in-hospital death and 1.55 (95%CI: 1.06-2.29; p=0.026) for death over the course of the 12 month follow-up. Probability of survival according to referral to palliative care is shown in figure 1.

## DISCUSSION

Decision making regarding referral to and implementation of exclusive palliative care in older patients is a complex issue. While one in every four acutely ill older adults admitted to geriatric wards is referred to palliative care, there is little consensus on how to best assess and communicate prognosis in geriatric populations. The establishment of better clinical guidelines may support early recognition of inpatients, who could benefit from palliative care, and provide critical information to patients and caregivers involved in the decision-making process.

Given current palliative care guidelines are often focused on oncologic patients, clinicians may overlook other aspects of significance to geriatric care. In fact, most older adults are hospitalized due to non-neoplastic conditions such as dementia and cardiovascular or respiratory diseases.^(^ ^23^ ^)^ In this study, male inpatients, inpatients suffering from cancer or advanced dementia and those with low albumin levels were more likely to be referred to exclusive palliative care. Two out of every three palliative care patients died within 12 months of hospital admission, mostly in hospital.

Five primary criteria for palliative care screening upon hospital admission have been proposed in a 2011 consensus, as follows: expected survival under 12 months, frequent hospital admissions, recurrent physical or psychological symptoms, complex care requirements, functional dependency and failure to thrive.^(^ ^24^ ^)^ However, this proposal was not aimed at older adults. The significance of factors other than those typically seen as indicative of potential palliative care eligibility was highlighted in this study. The contribution of nonneoplastic comorbidities to palliative care eligibility in acutely ill older adults was also confirmed: apart from cancer, advanced dementia, congestive heart failure, chronic kidney disease, chronic obstructive pulmonary disease and cirrhosis were all major reasons for referral to palliative care.^(^ ^23^ ^)^

### Predictors of referral to exclusive palliative care and mortality

In a systematic review by Coventry et al., several general and disease-specific characteristics have been described as potential predictor variables, and could help clinicians in referral of older adults to palliative care. However, these prognostic models were still thought to be inaccurate.^(^ ^25^ ^)^

In this cohort, male sex, advanced dementia and low albumin levels were independent predictors of exclusive palliative care eligibility and were associated with significant clinical complications, such as delirium, infections and pressure ulcers. The recognition of factors potentially related to referral to exclusive palliative care, and confrontation of such factors with predictors of death in palliative care patients, are an important step towards selection of appropriate treatment strategies for older adults.

In this study, advanced dementia, low albumin levels, delirium and pressure ulcers were associated with increased in-hospital and 12 month mortality. Also, male patients were more likely to be referred to palliative care, even though gender was not a significant predictor of death. Gender-related issues have not been fully addressed in palliative care. In contrast with findings in this study, men are thought to be less willing to discuss their terminality compared to women.^(^ ^26^ ^)^ However, gender-based differences in pain and suffering experience and the impact of gender on patient-caregiver relationships require further investigations before any conclusions can be drawn. Cancer was also an independent predictor of referral to exclusive palliative care in this study, and was associated with in-hospital mortality, but not 12 month mortality.

### Sociodemographic aspects

Significantly higher in-hospital mortality in the palliative care group suggests that palliative care measures were introduced late in the course of illness in this cohort. Many patients would likely have benefited from earlier implementation of palliative care, particularly over the course of primary care follow-up.^(^ ^27^ ^)^ In a survey of two thousand cancer-related deaths by Beccaro et al., access to palliative care services was thought to be associated with sociodemographic characteristics of patients and caregivers, such as higher levels of education.^(^ ^28^ ^)^ This study was based on public hospital data and many patients in the sample relied exclusively on public health system resources. Lack of access to proper primary care may have therefore compromised timely implementation of preventive and curative measures and quality of life related actions.

### Family and caregiver involvement

Most families and caregivers participated in palliative care referral decision, but the number of patients involved or even aware of their own prognosis was limited. Although this does not necessarily mean that patient preferences are dismissed, it certainly emphasizes the influence of family members in their care, particularly in Latin American countries.^(^ ^29^ ^)^ In a study with 202 participants, Bullock et al., reported that cultural beliefs, values and communication patterns are significant factors in end-of-life decision making.^(^ ^30^ ^)^

Previous studies have also shown that many physicians consider full information sharing to be difficult, and patients often prefer not be fully informed about their own health, which might have contributed to our results.^(^ ^31^ ^)^

### Additional findings

Opioids were widely used in the palliative care patient management in this study. While important and often essential in this group of patients, opioids are also a potential cause of adverse events and should be used with caution.^(^ ^32^ ^)^ Conversely, terminal sedation was found to be limited to a few cases. This was not surprising, as terminal sedation is a last resource strategy for management of refractory symptoms and pain. However, the high number of patients with dementia associated with hypoactive delirium prior to death in this sample must also be taken into account.^(^ ^33^ ^)^

### Limitations

This study had several limitations. First, it is a singlecenter study involving a unit specialized in care of high complexity geriatric patients with limited access to proper primary care prior to admission. This may have limited generalizing results; but previous studies have also shown that sociodemographic factors may interfere with access to palliative care.^(^ ^28^ ^)^

Also, only medical records containing data on referral to exclusive palliative care were fully reviewed, which might have led to errors in classification. However, significant differences between palliative and nonpalliative care groups suggest such errors to have been minimum. Finally, the decision to refer patients to exclusive palliative care may have reflected failure of previous therapeutic strategies, which was not measured in this study. Likewise, palliative care-specific interventions were not investigated in detail, precluding discussions on opioid use and terminal sedation prescription at this stage.

## CONCLUSION

Along with cancer, advanced dementia is a major trigger of referral to exclusive palliative care; delirium, pressure ulcers and hypoalbuminemia are important predictors of death. Although few patients were under palliative care upon admission, many had been referred to palliative care by the end of hospital stay. Given hospitalized older adults may not have been screened for palliative care eligibility prior to admission, clinicians must be aware of factors indicative of poor prognosis, and take quick action to provide the best possible care for patients and respective caregivers and/or family members.
